# Safety profiling of technical lignins originating from various bioresources and conversion processes

**DOI:** 10.1016/j.heliyon.2024.e32131

**Published:** 2024-05-31

**Authors:** T. Jayabalan, P. Pandard, G. Binotto, J. Gomes, X. Ceschini, A. Aube, F. Gondelle, F. Pion, S. Baumberger, A. Jongerius, R.J.A. Gosselink, E. Cozzoni, G. Marlair

**Affiliations:** a Institut National de l'Environnement Industriel et des Risques (Ineris), Parc Technologique Alata, 60550 Verneuil-en-Halatte, France; b Institut Jean-Pierre Bourgin, INRAE, AgroParisTech, CNRS, Université Paris-Saclay, 78000 Versailles, France; c Avantium Chemicals B.V., Zekeringstraat 29, 1014 BV Amsterdam, The Netherlands; d Wageningen Food and Biobased Research, 6708 WG Wageningen, The Netherlands; e BEES Design, Via Bargellini n. 7, 50059 Vinci, Florence, Italy

**Keywords:** Technical lignins, physico-chemical characterization, thermal hazard, dust explosion risk assessment, biodegradability, safety profile influencing factors

## Abstract

In this work, a set of eight technical lignin samples from various botanical origins and production processes were characterized for their chemical composition, higher heating value, size distribution, dust explosion sensitivity and severity, thermal hazard characteristics and biodegradability, in further support of their sustainable use. More specifically, safety-focused parameters have been assessed in terms of consistency with relating physico-chemical properties determined for the whole set of technical lignins. The results emphasized the heterogeneity and variability of technical lignins and the subsequent need for a comprehensive characterization of new lignin feedstocks arising from novel biorefineries. Indeed, significant differences were revealed between the samples in terms of hazards sensitivity. This first comparative physico-chemical safety profiling of technical lignins could be useful for the hazard analysis and the safe design of the facilities associated with large scale valorisation of biomass residues such as lignins, targeting “zero waste” sustainable conversion of bioresources.

## 1 Introduction

The growing emphasis and transition from the use of fossil resources to renewable ones, as well as the advances in biorefinery research towards a circular economy, have paved the way for exploring innovating use of biomass residues. In particular, lignocellulosic feedstocks like lignins have already shown promising industrial value as renewable carbon resources for broad applications [1,2] as well as for very specialized usage [3,4]. Technical lignins are side streams historically originating from the pulp and paper industry, and more recently as recalcitrant residues from lignocellulosic conversion through so-called “second generation biorefineries”. The different combinations of plant feedstocks, lignocellulose transformation processes and down-stream treatments result in a wide range of available pilot-stage or commercial lignin samples with various structural and chemical characteristics [5]. Among these lignins are kraft lignins, lignosulfonates, soda and organosolv lignins, each corresponding to one type of pulping process. Historically, lignins were mainly valorised by combustion to produce energy in recovery boilers due their significant calorific value, and subsequently they have been economically considered for a long time as low-grade fuels [6]. Alternative promising applications were later identified ranging from adhesives, adsorbents or polymer composites. They were also considered as raw material for processing carbon nanofibers and more recently as biobased options for chemical conversion into a wide range of chemicals [7]. Research is being actively carried out to develop value chains in a biorefinery perspective to render them commercially viable and to potentially replace the fossil-based products with bio-sourced ones in the future [8].

The emerging development of lignin value chains in the context of the bioeconomy in so-called second-generation lignin biorefineries [9] should therefore be based on the principles of green chemistry and environment sustainability. Although safety has not been fully promoted as a recognized pillar of environmental sustainability [10], even if some metrics regarding safety performances are sometimes briefly discussed [11], there are clear cross-linking aspects binding industrial safety and social, economic and environmental aspect of the sustainable development concept, as clearly underpinning in the 12 principles of green chemistry. Sustainability cannot be achieved alone and failure to assess, identify and promote safety may reduce the sustainability of the process. Moreover, both industrial ecology and circular economy trend advocate the replacement of fossil-based resources with renewable materials, the valorisation of all process residues, the use of safer products (solvents, chemicals…) and the selection of inherently safer design of processes as pioneered by T. Kletz [12]. Therefore, safety plays an important role in the promotion of sustainable valorisation of technical lignins and must be carefully assessed on the full value chains explored in that context.

Safety should be considered at the early stages of process development so that corrective actions and alternate pathways may be investigated with minimal cost and ease for incorporation. The 1^st^ step in the safety assessment of lignin value chains is to evaluate the hazards related to the material streams, and most particularly the lignin feedstocks. Accessing full characterization of physico-chemical, health and environmental hazards of lignins would be a prerequisite, in particular for obtaining their “end of waste status” according to the EU’s waste framework directive 2008/98/EC.

Recent reviews consider technical lignins as safe raw materials bearing low toxicity [13] or even do not mention any safety aspects [7], which can be supported by some commercial use of some technical lignins in feed or deduced from early assessments coming from previous studies dealing with the potential of lignins in cosmetics [14]. However, some papers indicate possible toxicity of lignin-derived compounds [15,16,17]. Moreover, biomass-based materials under certain conditions are susceptible to entail risks related to dust explosion, self-heating and fire hazards, which need to be addressed during the early stages of process development. An inadequate storage and handling of biomass may result in fires and explosions possibly causing injuries to employees, sometimes loss of lives and considerable economic losses and potential environmental damage [18]. The flammability characteristics of biomass need to be determined individually due to their heterogeneity as they can show varied risk profiles [19]. As far as lignins are concerned, information on the safety characteristics is very limited and the ones available in databases do not provide complete characterization. For example, in the Gestis-Dust-Ex database, only 5 datasets relate to lignin or lignin containing samples, providing partial information of their combustibility and explosivity sensitivity. Moreover, this information cannot be fully related to key physico-chemical properties of the samples, such as size distribution, humidity, and impurity content, rendering hazardous the extrapolation of these mentioned safety characteristics to new lignins of interest. Besides, the explosion characteristics for one type of wood dust containing, as a woody material, significant proportion of lignin are reported to be 10 times worse than another wood dust found in the same Gestis-Dust-Ex database [20]. The reason for such a variation likely lies in factors such as material properties, particle size distribution, chemical composition, which are not systematically provided along with explosion hazard parameters.

Most of previous studies related to thermal behaviour of lignins have concluded on their moderate thermal stability, leading to significant thermal degradation, generally over 300°C and still leaving high residue content in thermogravimetry analysis (TGA) experiments. Above 300°C, charring degradation mode has been evidenced due to the presence of aromatic rings and cross-linking transformation under stress that can be turned into beneficial use of lignins and lignin-derivatives as fire retardant components in composite polymers. As regards the self-heating hazard and the explosion sensitivity of lignins, few scientific works have been performed to our knowledge. More specifically there is a lack of insights into the influence of the variable chemical nature and structure of technical lignins with varying physico-chemical properties among them. It shall however be reminded that according to the Globally Harmonized System (GHS), lignins are classified as “combustible dust” with the associated official ‘hazard and precautionary statement’: “may form combustible dust concentrations in air”. As a summary of the our survey of useful literature and to our knowledge, no dedicated work has been performed yet to assess the physico-chemical properties of technical lignins in terms of influencing parameters of the safety profiles of these biomass resources of growing commercial interest, although it is worth to mention the recent work focusing on the ageing effect of biomass dust that may influence in time the properties of technical lignins studied in our work in terms of accidental ignition sensitivity [21]. In addition, some research has also been made as regard the influence of lignin source on its pyrolysis behaviour [22], but no conclusion can be anticipated from that work as regard the safety aspect.

Therefore, the present work addresses these issues by providing experimental results on the physico-chemical properties and the safety characteristics of a set of eight lignin samples with diversified origins (wood-based and agricultural residues) and production process (acid, soda, Kraft and enzymatic processes). These samples were selected as representative of either pilot-stage or commercial biorefinery processes and analysed for their composition, physical characteristics and biodegradability, physical safety hazards being mainly addressed through the fire and explosion risks. The results are discussed in terms of lignin characteristics variability and risk level ranking, in the perspective of the development of sustainable second generation biorefineries.

## 2 Materials and methods

### 2.1 Lignin samples

Eight lignin samples (Table 1) were selected for this study. Wood acid lignins AHL-1, AHL-2 and AHL-3 were produced using the Dawn Technology process at the Avantium pilot plant in Delfzijl, the Netherlands and from the laboratory of Avantium in Amsterdam, the Netherlands [23]. The AHL samples were received in the form of powders and prills, which were further grounded to have powder in sufficient amounts for testing. The HFL sample was produced from wheat straw via steam explosion pre-treatment followed by enzymatic hydrolysis and was received as a wet cake (c.a. 50 wt% of water). Before utilization, the sample was dried under a fume hood at room temperature and ambient pressure, then manually milled and dried under vacuum to obtain a fine powder. The commercial soda lignin sample WSL was produced from a mix of wheat straw and sarkanda grass (Protobind 1000, supplied by GreenValue Enterprises LLC, USA). The commercial pine Kraft lignin sample PKL was supplied by Ingevity (USA). Two eucalyptus-based alkali treated lignins obtained from a Kraft process, designated as EKL-1 & EKL-2, were supplied from the H2020 EUCALIVA EU-funded project [24]. EKL-2 differs from EKL-1, as it had undergone an oxidation step at 80 °C for volatiles removal and subsequent acid and water washing steps.

**Table 1:** Lignin samples selected for the study

Sample designation	Biomass origin	Extraction process
HFL	Wheat straw	Hydrolysis fermentation-distillation
AHL-1	A mix of pine and mixed woods	Acid hydrolysis
AHL-2	A mix of pine and mixed woods	Acid hydrolysis
AHL-3	Pine wood	Acid hydrolysis
WSL	Wheat straw/sarkanda grass	Soda
PKL	Pine	Kraft
EKL-1	Eucalyptus	Kraft
EKL-2	Eucalyptus	Kraft process followed by oxidation step

### 2.2 Characterization methods

#### 2.2.1 Physico-chemical characterisation of lignins

##### 2.2.1.1 Humidity content

The moisture content of lignins was determined using a Mettler Toledo HX204 thermal desiccator. The samples were air-dried at a temperature of 110°C till the mass variation is stabilized. The moisture was calculated by differential weighing before and after drying.

##### 2.2.1.2 Particle size distribution

The particle size distribution of the lignin powders was determined using a laser granulometer Malvern 3000E following the ISO standard [25] used for the determination of particle size distribution. The sample to be analysed is dispersed beforehand in distilled water which is then subjected to a beam of monochromatic laser. The particle size distribution is expressed as a percentage by volume and number.

##### 2.2.1.3 Morphology

SEM analysis was carried out using FEI quanta-200 F to study the morphology of the lignin samples.

##### 2.2.1.4 Higher heating value

The determination of the higher heating value (HHV) was measured experimentally using an oxygen bomb calorimeter Model Parr 6100, based on ISO standard [26]. The higher heating value was calculated from the adjusted temperature increase and the effective heat capacity of the calorimeter, considering all the necessary corrections. The result provided is an average of 2 calorific value measurements.

##### 2.2.1.5 Elemental analysis

The organic microanalyzers used for the C, H, N and O analysis were designed and manufactured by the Instrumentation Department of the Institute of Analytical Sciences, Lyon.

A total combustion of the analytical sample at 1050 °C under a stream of oxygen was carried out. Carbon and hydrogen are transformed into carbon dioxide and water respectively and quantified by using specific infrared detectors for CO_2_ and H_2_O. CO_2_ and water from the combustion are trapped on ascarite and magnesium perchlorate. Nitrogen is transformed into various nitrogen oxides and further reduced to molecular nitrogen and quantified by a thermally conductive detector. Sulphur in the samples is converted to SO_2_ by complete combustion of the analytical sample at 1350°C under a stream of oxygen. and quantified using a specific infrared detector. For the oxygen analysis, a total pyrolysis of the analytical sample is carried out at 1080°C under a stream of nitrogen. Oxygen compounds from the pyrolysis is converted into CO by passage over activated carbon at 1120°C and the CO is quantified by a specific infrared detector.

##### 2.2.1.6 Total sugar content

Lignin samples were hydrolyzed by a two-step sulfuric acid hydrolysis starting with 12 M H_2_SO_4_ at 30 °C for 1 h followed by 1 M H_2_SO_4_ at 100 °C for 3 h and analysed according to a published procedure [27]. The hydrolysate obtained for each sample was neutralized by calcium carbonate until acidic pH as indicated by bromophenol blue. Resulting monosaccharides were separated and quantified by HPAEC-PAD on a Dionex Carbo-Pac PA1 column and precolumn under the following conditions: sodium hydroxide/water gradient at 35 °C; flow rate 1 ml min^-1^. Post column addition of 500 mM NaOH at a flow rate of 0.2 ml min^-1^ was used for amperometric (PAD) detection.

#### 2.2.2 Hazard characterization of lignins

##### 2.2.2.1 Explosion severity

The explosion severity was determined by reference to key driving parameters of the potential consequences and effects of an explosion, which include the maximum explosion pressure (P_max_), maximum rate of pressure rise (dp/dt)_max_ and dust explosion index (K_St_ Value). These parameters were measured/calculated in accordance with the relevant European standards (EN 14034-1 and EN 14034-2) [28]. Experiments were performed in a 20 L sphere apparatus supplied by Kuhner. The test chamber is a hollow sphere made of stainless steel with a cooling water jacket to dissipate the heat of explosion. The apparatus consists of a dust dispersion system equipped with valves and dispenser. The ignition system consists of 2 pyrotechnic igniters capable of delivering 5 kJ of energy each. The explosion pressure is recorded as a function of time using piezoelectric pressure sensors.

##### 2.2.2.2 Ignition sensitivity

The ignition sensitivity of a powder represents the probability that the powder would ignite when subjected to certain condition. 2 parameters characterizing ignition sensitivity, namely the Minimum Ignition Energy (MIE) and the Minimum Ignition Temperature in Layer (MIT_L_) were evaluated experimentally.

##### 2.2.2.3 Minimum Ignition Energy - MIE

The test was conducted based on the ISO standard [29]. The minimum ignition energy (MIE) is the minimum amount of energy required to ignite a dust mixture with air under specified test conditions. The MIE was measured using MIKE 3 apparatus supplied by Kuhner and consists of a vertical cylindrical glass tube with a volume of 1.2 L. The dust sample is pneumatically dispersed within a vertical tube opened at the top at a pressure of 7 bar. The ignition is provided by an electric spark initiated within the cloud between two electrodes separated by a 6 mm gap. The criterion considered for rating the lowest ignition temperature as the MIE is the observation of a flame propagation which is performed visually by the operator.

##### 2.2.2.4 Minimum Ignition Temperature in layer (MIT_L_)

The MIT of a dust layer is defined as the lowest temperature of a hot plate at which the ignition of a dust layer of a specified thickness occurs. The practical thichness under consideration is usually 5 mm. The test was carried out in accordance with the existing standards [30]. This method refers especially to industrial equipment with hot surfaces where dust create layers of different thickness can build up and trigger danger under contact with air.

The test apparatus supplied by ANKO consists of a metal plate having a working surface of 21.7 cm in diameter and a thickness of 1 cm. The plate is electrically heated, and its temperature controlled by a device whose sensor is a thermocouple mounted in the plate, near the centre and whose junction must be 1 mm ± 0.5 mm from the upper surface and in good thermal contact with the plate. A similar thermocouple should be mounted in the same manner near the control thermocouple and should be connected to record the temperature of the surface during the test.

##### 2.2.2.5 Thermogravimetry analysis (TGA)

**Figure 1:**
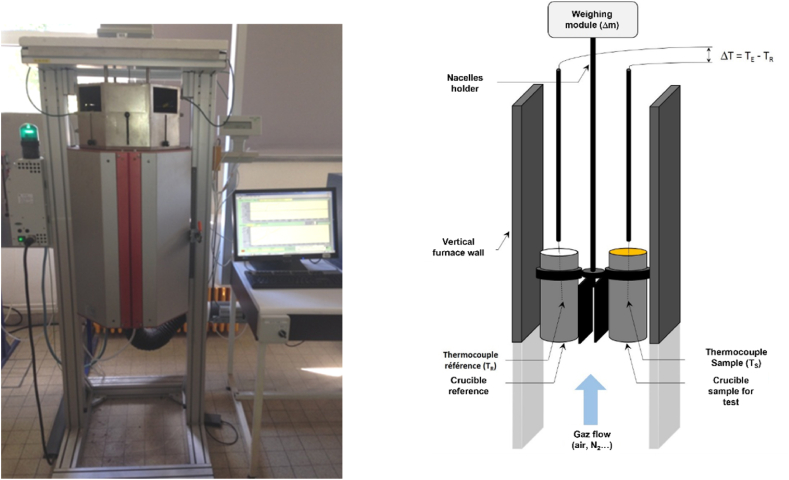
Large scale TGA-DTA instrument setup developed at INERIS

Thermal analysis of the lignin samples was carried out using a purpose-built instrument (Figure 1) allowing the application of test method [31]. The results can be used for assessing thermal hazards of solid samples. This equipment is working with a sample mass of 1 g or more, largely over the few milligrams range used in the conventional laboratory scale TG-DSC commercial instruments, which subsequently allows its use as a screening test for self-heating assessment.

About 1 g of test sample is placed in a small container made from wire mesh; an inert reference substance alumina is placed in an identical container. Both containers are introduced at ambient temperature into an oven and heated at a fixed rate (5°C) in an air flow rate of 140 ml.min^-1^. The microbalance has a precision of ± 0.1 μg and the samples and the temperature range from 25°C to 800°C under air.

##### 2.2.2.6 Assessment of the ultimate biodegradability of lignin samples

The determination of the biodegradability of the lignin samples was carried out according to OECD 301F test method [32] (manometric respirometry test). In this test, the biodegradation of organic compounds is measured in an aqueous medium by determining the oxygen consumption in a closed respirometer. The test mixture contains a mineral medium, the organic compound as the sole source of carbon and energy (around 100 mg ThOD/L) and a mixed inoculum obtained from a waste-water treatment plant (WWTP) receiving predominantly domestic sewage. The CO_2_ released is absorbed by concentrated NaOH and manometrically measured as a negative pressure. The amount of oxygen taken up by the microbial population during biodegradation of the test substance (corrected for uptake by blank inoculum, run in parallel) is expressed as a percentage of theoretical oxygen demand (ThOD). This test is suitable for both soluble and poorly soluble compounds. In this test, a chemical is considered being readily biodegradable if the pass level (60% ThOD) is reached in a 10-day window within the 28-day period of the test.

The test duration (normally 28 days) has been extended to 60 days in accordance with the recommendations of the ECHA guidance document "Guidance on Information Requirements and Chemical Safety Assessment - Chapter R.7b: Endpoint specific guidance", relating to enhanced biodegradation screening tests, as some biodegradation was observed but did not reach a plateau by the end of the usual test duration, i.e., after 28 days.

A reference chemical: aniline, which meets the criterion for ready biodegradability (i.e., percentage degradation > 60% by day 14), has been included to each experiment in order to check the successful completion of the test procedure.

Two successive experiments were carried out due to the experimental conditions applied and the experimental devices available. The test conditions applied are summarized in Table 2. In each experiment, three replicates have been carried out for each lignin sample. A third experiment was performed to confirm the results obtained for HFL and to check that the degradation observed could effectively be attributed to the activity of microorganisms.

**Table 2:** Tests conditions applied during the biodegradation tests

	Lignin	DThO NH_3_ (mg/mg)	Tested concentration (mg ThOD/L)	Inoculum concentration (mg/L)
1^st^ exp.	AHL-1	1.778	95.8	27
	AHL-2	1.879	110.0	
	HFL	1.538	75.7	
	WSL	1.871	97.0	
2^nd^ exp.	EKL-1	1.863	96.0	21
	EKL-2	1.558	94.3	
	HFL	1.538	92.1	
	AHL-3	1.894	93.4	
	PKL	1.847	94.5	
3^rd^ exp.	HFL	1.538	92.1	27

For sufficiently soluble compounds, the degree of biodegradation was also estimated by comparing the TOC concentration at the end of the incubation period with the theorical organic carbon concentration introduced the test vessels.

## 3 Results and discussions

The detailed characterization of these 8 technical lignins has included particle size distribution, moisture content, elemental analysis, quantification of residual sugars, heating value, SEM analysis and imaging of the sample morphology. Indeed, all these parameters may significantly influence the physico-chemical and environmental hazard profiles of lignins. Safety focused characterization has mainly addressed the fire and explosion risk, through consideration of self-heating hazard, a common hazard of any organic powdered material as well as the dust explosion hazard, an often-underscored issue in the agro-industries in the past that had caused many casualties in several dramatic incidents in the last decades. Some consideration of biodegradation of lignins was also done, providing useful information toward efficient use or elimination route.

### 3.1 Physico-chemical characteristics of the lignin samples

#### 3.1.1 Particle size distribution

**Figure 2:**
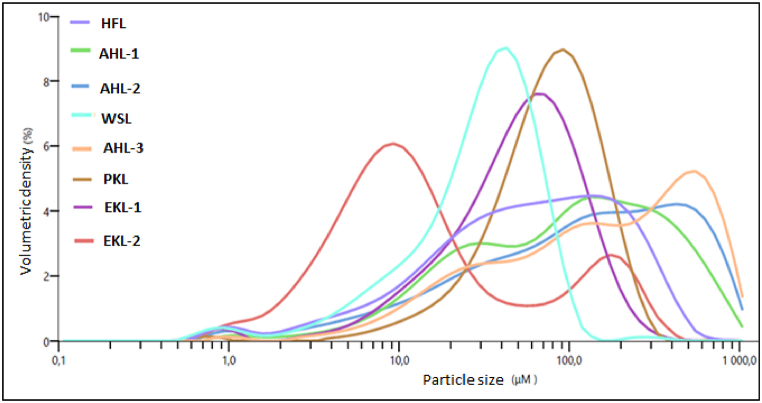
Particle size distribution curves for the different lignin samples.

Key parameters describing the particle size distribution for the different lignin samples are gathered in Table 3 and overall graphical comparison of samples showing the resulting variety in terms of particle size distribution profiles is presented in Figure 2. The Dv(50) which is the median particle size, the Sauter diameter D(3,2) or the surface weighted mean which is the ratio of the volume to surface area were analysed. Additionally, to get a more complete description of the particles, the distribution of smallest particle fraction of diameter less than 500 μm was further specified by quantifying Dv(10) and Dv(90) size distribution parameters which respectively represent the diameter below which 10 % and 90 % of the cumulative mass fraction is retrieved.

**Table 3:** Results of the particle size distribution and specific surface area for the different lignin samples.

Sample	Dv(10) (μm)	Dv(50) (μm)	Dv(90) (μm)	Sauter diameter D(3,2) (μm)	Fines particles < 500 μm (%)
HFL	6,7	38,5	161,0	16,5	88,6
*Std. Dev.*	*0,4*	*3,3*	*26,6*	*0,2*	*5,5*
AHL-1	13,7	94,0	446,0	28,6	92,1
*Std. Dev.*	*0,6*	*15,6*	*41,1*	*2,6*	*1,3*
AHL-2	12,2	131,0	597,0	23,5	85,3
*Std. Dev.*	*0,6*	*1,5*	*6,5*	*0,9*	*0,4*
AHL-3	17,4	141,0	641,0	41,5	82,6
*Std. Dev.*	*0,8*	*14,3*	*36,7*	*2,7*	*2,5*
WSL	7,8	32,6	69,3	13,7	100,0
*Std. Dev.*	*2,5*	*11,8*	*20,8*	*3,8*	*0,0*
PKL	30,0	92,0	193,0	38,6	99,7
*Std. Dev.*	*2,1*	*4,9*	*83,0*	*3,1*	*0,4*
EKL-1	15,3	68,2	159,0	20,0	99,8
*Std. Dev.*	*1,3*	*7,3*	*17,1*	*1,1*	*0,3*
EKL-2	3,2	11,3	71,5	7,2	99,7
*Std. Dev.*	*0,2*	*0,7*	*4,0*	*0,3*	*0,3*
« Lignin »(a)	24,4	80,9	204	49,1	NA

(displayed values from 3 measurements by sample (not known for “Lignin” sample); Sdt. Dev. Stands for standard deviation)

(a) Not tested in this work, published data from lignin sample of unknown origin for comparison [33]

The results show that all the lignin samples have a median size of less than 150 μm and 85 to 100 % of the particles were found to be less than 500 μm. The analysis also reveals significant differences from one sample to the other in terms of overall particle size distribution profile. The samples EKL-1, PKL and WSL show unimodal distribution, whereas EKL-2 shows a bimodal distribution and both AHL samples present a multimodal distribution. The Dv(10), Dv(50) and Dv(90) values confirm a wide distribution range of the particle sizes for the AHL and HFL samples. Particulates and powders smaller than 500 microns (μm), are considered to have the potential to present a flash-fire hazard or explosion hazard when suspended in air [34]. Smaller particles are in general more easily dispersed and will stay suspended for a longer duration and easily heated to the point of devolatilization and therefore easily ignited and can better propagate a flame [35]. Given the complexity in the particle size distribution and the heterogeneity of structures, it is more important to monitor the proportion of fines present as it basically influences the reactivity. Recently, more fundamental work making use of the Godbert-Greenwald apparatus, an open vertical combustion tube and the 20-litre sphere has also confirmed the underpinning complexity of biomass dust explosion and relating interacting parameters. These parameters are playing a role well beyond to particle size distribution which justifies the need of very accurate determination of physicochemical properties of biomass derived dusts. All biomass powder explosions as a matter of fact reveal to be hybrid explosions (gas + solid matter), due to the initial pyrolysis stage which often constitutes the limiting factor in the overall process leading to the explosion of a combustible dust in mixture with air [36].

#### 3.1.2 Morphology of the lignin samples

As reported quite recently [21], technical lignins, in the form of biomass more or less finely divided particles, is likely to behave in terms of risk of dust explosion, as a “non-traditional dust”, since liable to differ in terms of shape and morphology from the conventional spherical-like particles for which most of dust explosion phenomenon knowledge has been achieved so far. Therefore, SEM imaging was considered as an important aspect of our work to further assess how far technical lignins studied may vary from this endpoint. Another key interest of SEM imaging is the potential observation of particle agglomeration phenomenon, another key aspect in organic dust explosion risk evaluation [37].

**Figure 3:**
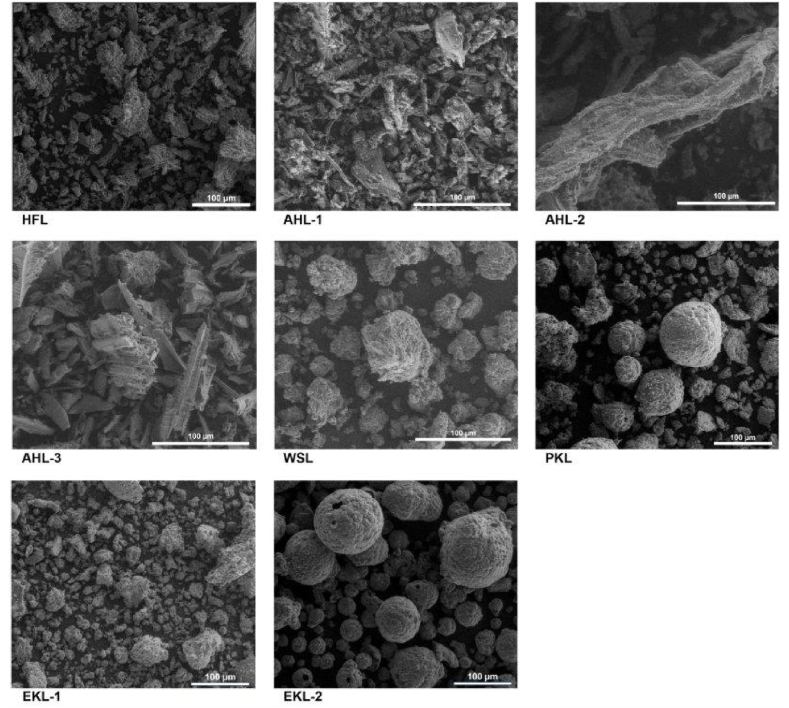
SEM images of the lignin samples

The SEM images of the lignin samples are gathered in Figure 3 as an illustration of the varying morphology of the studied lignins. Indeed, as can be seen, the lignin samples exhibit strongly variable morphological features ranging from irregular, fibrous, spherical to needle-shaped particles. The HFL sample show irregular flake like structures and the AHL samples are more fibrous and exhibit needle like structures. The particles of the samples WSL, PKL and EKL-2 reveal spherical shaped structures. The SEM images do not reveal agglomeration of particles in the test lignin samples. This phenomenon is known to be dependent on the moisture content, particle size and its distribution and shape. Finer dust and higher moisture content are favoring factors towards particle agglomeration and subsequently can modify the ignition behaviour [38].

**Figure 4:**
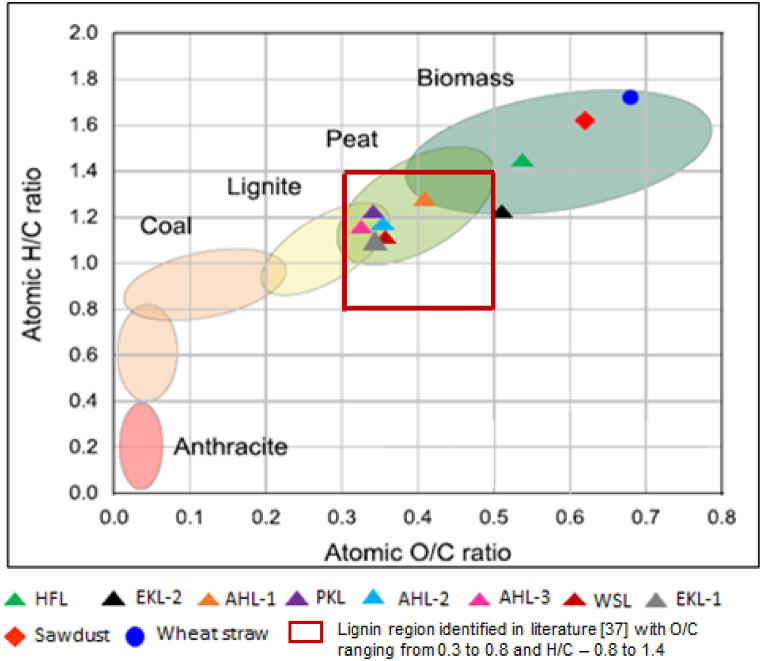
Van-Krevelen diagram for the comparison of lignin samples

#### 3.1.3 Elemental composition

**Figure 5:**
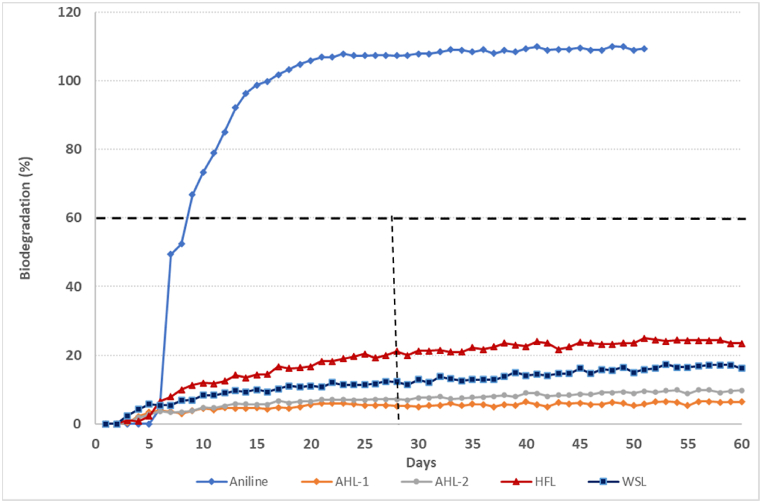
Biodegradation curves (1^st^ experiment)

The elemental analysis of the investigated lignin samples is reported in Table 4. The experimental relative error in the determination of the chemical composition of lignin samples was found to be less than 5%. The carbon content in the lignin samples was found to range between 48% to 60 %. Oxygen content varied from 28 to 37 %, hydrogen content reached 5 to 6 %, while sulphur was found in limited quantities ranging from 0 to 2 %. Only trace quantities of nitrogen were detected. To further analyse the results, the H/C ratio and O/C ratio of the lignin samples are plotted in the Van Krevelen diagram [39,40] as shown in Figure 5. The plot displays the variation of the H/C and O/C ratios of the lignin samples, showing that the samples HFL and EKL-2 have the highest O/C ration, greater than 0.5, whereas the other samples were found to present O/C ratios in the range of 0.35 to 0.4. The high elemental O content in the HFL sample may be partly due to the presence of the cellulosic oxygen content of the biomass fractions as they might not be fully isolated [41]. In the case of the EKL-2 sample, the extra oxidation step at 80 °C likely explains the increase in the oxygen content as compared to EKL-1 sample.

**Table 4:** Elemental composition and moisture content of the lignin samples (Elemental analysis data given are the mean values for two experimental determinations)

Sample	C (%)	H (%)	N (%)	O (%)	S (%)	O/C molar element ratio	H/C molar element ratio	moisture content (wt %)	Total carbohydrate content (%)
**HFL**	51.20	6.20	1.10	37.00	0.23	0.54	1.45	7.90	38.70
**AHL-1**	58.30	6.20	0.17	32.09	0.26	0.41	1.26	4.20	21.82
**AHL-2**	60.60	6.10	0.11	29.25	0.26	0.36	1.20	8.40	10.94
**WSL**	61.70	6.03	0.50	29.08	0.69	0.35	1.16	6.60	2.00
**AHL-3**	60.44	6.13	0.15	28.53	0.53	0.35	1.20	3.00	0.30
**PKL**	60.00	6.10	0.90	28.9	1.82	0.36	1.20	5.50	1.40
**EKL-1**	61.30	6.00	0.57	28.85	1.61	0.35	1.15	4.00	1.70
**EKL-2**	48.84	4.96	0.11	33.32	1.80	0.51	1.20	5.70	1.50

Two additional biomass samples with useful characteristics found in the open literature, namely those designated as sawdust and wheat straw were added to complete our analysis of lignin elemental composition through the use of the Van Krevelen diagram [18]. Samples HFL and EKL-2 are situated in the biomass region whereas the other samples showed O/C and H/C similarly to lignite and peat like materials (Figure 4). The differences could be mainly attributed to the degree of carbonization and degradation during chemical and thermal pre-treatments and reactions in the lignin pre-treatment and isolation process. The order of magnitude of O/C and H/C ratios of the lignin samples correspond to the values found in literature [39].

#### 3.1.4 Humidity

The moisture content of the lignin samples varied from 3 % to 8.4 % by weight. Dry powders are more susceptible to form an explosible dust due to the ease in the suspension in air and reduced tendency to form agglomerates.

#### 3.1.5 Total carbohydrate content

The results of the total carbohydrate content of the lignin samples are gathered in Table 4. The carbohydrate content of the lignin samples varied between 0.3 to 38.7%, these variations reflecting the different fractionation severity factors and removal rates of the (hemi)cellulosic fractions attributed to the process conditions and scales. The two pilot plant samples (AHL-1 and AHL-2) exhibited higher sugar contents than AHL3, which was consistent with the fact that they corresponded to earlier development stages of the acidic hydrolysis process of the biomass. Advantageously, these three samples allowed to cover a wide range of carbohydrate contents (0.3-22%) for a given type of lignin. The highest carbohydrate content was observed for HFL, in agreement with the fact that enzymatic hydrolysis leaves some recalcitrant cellulose in the solid residue [42]. In contrast, all the samples produced in alkaline conditions (EKL1, EKL2, PKL, WSL) exhibited low carbohydrate contents (lower than 2%), due to more selective lignin isolation and enhanced purification processes.

#### 3.1.6 Higher Heating Values (HHV)

The experimental HHV of the lignin samples varied from 20 to 25 MJ/kg (see Table 4), in accordance with literature data [43]. The HFL and EKL-2 samples showed lower heating values compared to the other samples. It was observed that the energy content decreased inversely to relating O/C ratio, which confirmed previous results obtained on other technical lignin samples [44].

The theoretical HHV were further estimated from well-known predictive empiric models (see eq. 1 to Eq3), based on the elemental composition of the lignin samples.

(eq 1) Boie equation [45]: HHV = 0.3517(C) + 1.1625(H) - 0.1109(O) + 0.1047(S) + 0.0628(N)

(eq.2) Mendeleev equation [46]: HHV = 0.339 (C) + 1.256 (H) - 0.109 (O) + 0.109 (S)

(eq. 3) Ozyuguran equation [47]: HHV = 0.3880 (C) + 0.4022 (H) - 0.0354 (O) + 0.1987 (N)

Boie equation (eq.1) was originally developed as an empirical correlation to measured heating values of solid fuels and its use was extended at later stage for a large panel of samples which included biomass fuels, coal, coke char, shale oils and coal. Also used for estimation of heating values of plastics, Boie correlation has revealed relatively robust even outside its original domain of validation [48,49]. Mendeleev equation (eq.2) was developed for any fuels and Ozyuguran equation (e.3) developed specifically for biomass materials. For all the lignin samples, good agreement was observed between the experimental and the calculated values (Table 5), with an average difference of 0.4% on all the samples.

**Table 5:** Experimental and calculated HHV for the tested lignin samples a) Data as mean value from two experimental determination

Sample	HHV experimental results (MJ/kg)^a^	Calculated HHV from Boie equation (MJ/kg)	Calculated HHV from Mendeleev equation (MJ/kg)	Calculated HHV from Ozyuguran equation (MJ/kg)
**HFL**	20.67	21.23	21.14	21.27
**AHL-1**	23.93	24.22	24.08	24.01
**AHL-2**	25.2	25.26	25.10	24.96
**WSL**	25.30	25.61	25.40	25.43
**AHL-3**	25.02	25.25	25.07	24.92
**PKL**	25.32	25.26	25.05	24.90
**EKL-1**	25.58	25.56	25.35	25.28
**EKL-2**	20.34	19.50	19.35	19.78

### 3.2 Hazard profiling of Lignins

#### 3.2.1 Explosion severity

The dust explosion severity of the lignin samples was evaluated through three parameters (see Table 6), namely the maximum explosion pressure (P_max),_ the maximum rate of pressure rise ((dP/dt)_max_) and the dust explosion index (K_st_). The K_st_ value was derived from the maximum rate of pressure rise (dP/dt)_max_ and the volume V of the explosion vessel according to the following equation:

K_st_ = (dP/dt)_max_ · V^(1/3)^ , with V = 20L

**Table 6:** Experimental results of explosion severity parameters of lignins

Sample	Max explosion pressure (Pmax) bar	Max. rate of pressure rise (dP/dt)max (bar/s)	Product specific constant - Kst (m.bar/s)	Explosion violence St class
**HFL**	8.5(±10%)	555 (±12%)	151((±12%)	St-1
**AHL-1**	8.8(±10%)	819(±10%)	222(±10%)	St-2
**AHL-2**	7.9(±10%)	801(±10%)	217(±10%)	St-2
**AHL-3**	8.1(±10%)	779(±10%)	211(±10%)	St-2
**WSL**	8.9(±10%)	905(±12%)	246(±12%)	St-2
**PKL**	8.5(±10%)	645(±10%)	175(±10%)	St-1
**EKL-1**	8.7(±10%)	763(±10%)	207(±10%)	St-2
**EKL-2**	7.7(±10%)	531(±12%)	144(±12%)	St-1
*Lignin a*	*∼ 6*	*∼ 560*	*152*	*St-1*

Lignin a: Kst value determined from data of Liu et al. [33], refer to note of table 3.

Based on the Kst values the explosion violence could be qualified into 4 classes (St) namely:

• K_st_ =0 : St-0, non-explosible
• 0 < K_st_ < 200 : St-1, low explosion violence
• 200 < K_st_ < 300 : St-2, medium explosion violence
• K_st_ > 300 : St-3, strong explosion violence

The P_max_ values for the lignin samples varied from 7.7 bar for EKL-2 to 8.9 bar for WSL. The (dP/dt)_max_ values increased from 531 m.bar/s for EKL-2 to 905 m.bar/s for WSL. The experimental error for Pmax and (dP/dt)_max_ values was found to be 10 %. The results show that 3 samples namely HFL, PKL and EKL-2 produced low explosion violence, whereas the other samples produced medium explosion violence, ranking these samples into St-2 severity class. It should be noted that incidents of explosion have occurred even with St-1 materials (e.g. Imperial sugars, USA) and should therefore not be neglected [50].

The results show that the lignin samples are susceptible to produce an explosion when dispersed in air ranging from low to medium explosion violence. The severity results for an explosion are regularly adopted for designing explosion-relief, explosion suppression and venting systems.

The dust explosion characteristics is strongly influenced by the moisture content as it plays a role in the agglomeration of the solids decreasing its dispersibility. The influence of moisture content on the explosibility characteristics is illustrated in Table 7 by varying the moisture content of the HFL lignin from 8% to 0.6 %. Drying increased the explosion severity by 21 % (in terms of (dP/dt)_max_) showing that dry lignins may be highly sensitive to explosion risks compared to lignins with higher humidity content. In the studied HFL sample case, according to confidence intervals regarding Kst Values obtained, explosion violence St class changes from St-1 to St-2 due to severe decrease in moisture content.

**Table 7:** Explosibility parameters of HFL sample with varying humidity content.

Sample	Moisture content	Pmax (bar)	(dP/dt)_max_ (bar/s)	Kst (m.bar/s)
HFL	8 %	8,5(±10%)	555(±12%)	151(±12%)
HFL	0,6 %	9,2(±10%)	676(±12%)	184(±12%)

#### 3.2.2 Ignition sensitivity

The parameters relevant for the assessment of the likelihood of occurrence of a dust explosion are essentially MIE and MITL values (see Table 8).

**Table 8:** Experimental results of the minimum ignition energy and temperature of the lignin samples.

Sample	Minimum Ignition Energy (MIE)	Minimum Ignition Temperature in Layer (MIT_L_) °C a)
**HFL**	30 mJ < MIE < 100 mJ	280
**AHL-1**	3 mJ < MIE < 10 mJ	300
**AHL-2**	3 mJ < MIE < 10 mJ	270
**AHL-3**	3 mJ < MIE < 10 mJ	260
**WSL**	3 mJ < MIE < 10 mJ	400
**PKL**	30 mJ < MIE < 100 mJ	> 410
**EKL-1**	10 mJ < MIE < 30 mJ	> 410
**EKL-2**	MIE > 1000 mJ	> 410

a) Determined from 3 trials at each tested temperature up to ignition occurrence

The MIE results displayed in Table 8 for the lignin samples show varying trends that range the samples in terms or ignition sensitivity as very critical for 4 out of the 8 lignin sample tested, (with values of 3 mJ < MIE < 10 mJ) to least sensitive ones (with 1 out of 8 lignin samples presenting a MIE value > 1000 mJ).

The experimental results of the MITL show that there was no ignition observed till 400 °C for the samples PKL, EKL-1 and EKL-2 which is the maximum temperature to be heated according to the standard and temperatures of 410°C were reached for the experiments. The samples AHL and HFL samples showed lower ignition temperatures in layer (measurements gave values lower than 300 °C). Some hot surfaces of equipment can therefore become an ignition source for the lignin dusts. According to EN 60079-14, the maximum surface temperature of the equipment shall not exceed a value of 75 K below the minimum ignition temperature of 5 mm layer of dust.

### 3.3 Thermal properties

The screening results from the TGA analysis may be helpful to categorize the products, according to the specific experience-based self-heating hazard rating and decision logics developed by Ineris towards further evaluation of the detailed self-heating risk profile:

− The most reactive samples (Class A, T < 250 °C), for which the test in an insulated oven must be carried out for all conditions of storage,
− The average reactivity samples (Class B, 250 °C < T < 400 °C), which may be tested in an insulated oven, if necessary (storage of large dimension, hot stored product),
− The least reactive samples (Class C, self-heating temperature greater than 400 °C), where no further testing is required.

The results of the pre-screening tests indicated that nearly all lignin samples belong to class A or class B (Table 9), meaning they are sensitive to self-heating during bulk storage except WSL lignin sample which falls under the category of class C. In the case of large-scale storage of lignins, it is therefore advisable to further qualify the actual self-heating risk of lignins by carrying out isothermal basket tests (oven tests) to check for the critical dimensions of storage and relating maximum safe storage duration in given operational conditions.

**Table 9:** Experimental results of pre-screening self-heating temperature of the lignin samples.

Sample	Pre-screening self-heating temperature °C	Classification
**HFL**	269	Class B
**AHL-1**	204	Class A
**AHL-2**	204	Class A
**AHL-3**	198	Class A
**WSL**	455	Class C
**PKL**	238	Class A
**EKL-1**	211	Class A
**EKL-2**	196	Class A

### 3.4 Biodegradability

**Figure 6:**
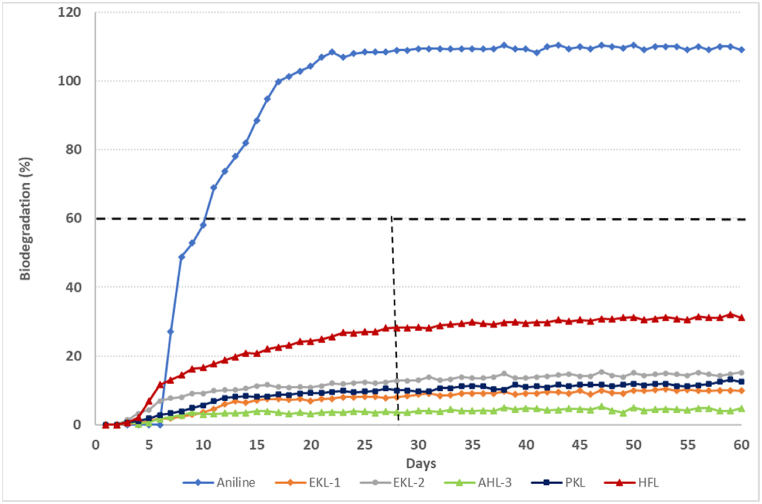
Biodegradation curves (2^nd^ experiment)

The results of biodegradability tests are summarized in Table 10, whereas Figure 5, Figure 6 display the biodegradation profiles of the tested lignins. For most of the lignin samples, no aerobic biodegradation was observed (it is commonly accepted that biodegradation has started when it exceeds the 10% level), whatever the lignin production process. HFL only exceeded 20% biodegradation after 28 days (1^st^ experiment: 21.3%; 2^nd^ experiment: 28.3%; 3^rd^ experiment: 24.5%). Two other lignin samples passed the 10% level after 28 days, *i.e.,* WSL and EKL-2.

**Table 10:** Biodegradation percentages obtained after 28 and 60 days

	Lignin	Biodegradation percentage (after 28 days)^a^	Biodegradation percentage (after 60 days)^a^
1^st^ exp.	AHL-1	5.0%	5.8%
	AHL-2	7.5%	9.5%
	HFL	21.3%	23.8%
	WSL	12.9%	17.1%
2^nd^ exp.	EKL-1	8.8%	10.6%
	EKL-2	12.9%	14.8%
	HFL	28.3%	32.1%
	AHL-3	4.0%	4.6%
	PKL	9.7%	13.4%
3rd exp.	HFL	24.5%	26.2%^b^

a : average of the three test vessels;

b : experiment stopped after 50 days.

The biodegradation rate of aniline (reference chemical), revealed to be higher than 60% (1^st^ experiment: 99.9%; 2^nd^ experiment: 94.8%; 3^rd^ experiment: 63.6%), within the first two weeks, hence confirming the validity of the results for the three genuine tests performed.

Whilst biodegradability of lignins if sometimes claimed considering lignin originates as a native constituent of lignocellulosic biomass [51], none of the technical lignin samples reveals as readily biodegradable in this stringent screening test, whatever the process and the source of biomass. These results, obtained with non-specific microorganisms from predominantly domestic WWTP, are consistent with the previous work [52] on a commercial alkali lignin, even though that lignins have been shown to be biodegradable by some specific bacteria and white-rot fungi [53,54,55]. HFL was the only lignin for which the biodegradation rate exceeded 20% biodegradation after 28 days. The extension of test duration up to 60 days did not significantly modify the biodegradation rate of the lignin samples, despite of test duration prolongation that had given more time to the microorganisms for accessing and degrading the test chemical. Additional experiments set up for lignin samples that not reached 10% biodegradation (i.e., lignin sample + reference chemical: aniline) showed that lignin samples at the concentration used in the test were not inhibitory towards the microorganisms from the WWTP.

For HFL, the results were consistent across the three experiments. The concentration of Total Organic Carbon removed (corrected for that in the blank inoculum control) at the end of the experiment was about 45% of the concentration initially present, confirming the biodegradation observed (exp 1 and 2). No lag phase was observed for degradation of HFL indicating that the biodegradation started quickly in the test vessels after contact between inoculum from WWTP and the lignin sample. The comparison of test conditions applied in the 3^rd^ experiment (HFL with inoculum; HFL without inoculum; HFL without inoculum + sterilising agent) confirmed that the degradation observed could effectively be attributed to the activity of microorganisms. Indeed, no biodegradation has been observed for the two test conditions without inoculum.

The first part of the degradation curve for HFL is typical of the biodegradation of a readily biodegradable compound even if the plateau phase is reached quickly. The high concentration of carbohydrates (mainly cellulose) in the tested sample of HFL (see table 4) could explain the biodegradation observed, as cellulose is being recommended in some biodegradation tests as reference substance.

## 4 General discussion and conclusions

In this study, eight lignin samples from four types of extraction processes had been selected, as representative of the most frequent lignin feedstocks accessible today or in near future, either commercially or as emerging from pilot or demo plants. The physico-chemical analyses of the samples showed that they covered a wide range of compositions, morphologies, and thermal properties, thus providing a relevant panel to perform a hazard profiling and biodegradability assessment of technical lignins. As summarized in Table 1, the samples exhibit different profiles as a function of both the lignin type and process conditions. The HFL sample turned out the most biodegradable one and exhibited the lowest HHV and explosion severity. These characteristics could potentially result from its fibrous structure and high carbohydrate content. In contrast to HFL, the two kraft lignin samples PKL and EKL1 were typified by their low carbohydrate contents, spherical morphologies and high HHV. The two samples showed similar hazard profiles (no ignition until 410°C, high self-heating risk) and very low biodegradability (14% after 60 days). Thus, the wood origin (Eucalyptus *vs* pine) did not affect the properties investigated here. Though WSL showed overall similar characteristics and profiles as PKL and EKL1, their explosion severity and self-heating risk were more pronounced, which may tentatively be assigned to their lower particle size. The comparison of the three AHL samples shows that the decrease in carbohydrate content did not affect the properties of the lignins apart from a slight decrease of the self-heating temperature. The three samples showed a lack of biodegradability and higher self-heating risks than the others, which suggests that specific chemical modifications take place upon the acid hydrolysis treatment. Finally, comparison of EKL1 and EKL2 indicated that the difference in the production process (mainly oxidation step) led to smaller lignin particles (5-times lower diameter) and improved the safety profile of the lignin (lower explosion severity) and its biodegradability. Taken together, these results demonstrate that it is not possible to draw general conclusions and predict the safety profile of lignins on the sole basis on their production process and physico-chemical characteristics. Safe use of lignins as renewable feedstocks in modern biorefineries is conditioned to appropriate assessment of fire and explosion hazard that in turn must be related to ad hoc characterization of physicochemical properties of lignin streams exploited. To that purpose, the results of our work, detailing the hazardous profile of a wide range of accurately characterized lignins may serve as useful reference data for advanced lignin biorefining.

## Data availability statement

Most data used in this article readily available in the paper and relating supporting information provided. Due to the nature of the research, and legal constraints applying, further supporting data are not available.

## CRediT authorship contribution statement

**T. Jayabalan:** Writing – original draft, Supervision, Resources, Project administration, Methodology, Investigation, Funding acquisition, Formal analysis, Conceptualization. **G. Binotto:** Writing – original draft, Validation, Investigation, Data curation, Conceptualization. **X. Ceschini:** Validation, Resources, Formal analysis, Data curation. **F. Gondelle:** Investigation, Data curation. **S. Baumberger:** Methodology, Formal analysis, Data curation. **R.J.A. Gosselink:** Methodology, Formal analysis, Data curation. **G. Marlair:** Methodology, Formal analysis, Data curation, Writing – review & editing.

## Declaration of Competing Interest

The authors declare that they have no known competing financial interests or personal relationships that could have appeared to influence the work reported in this paper.
