# Supplementary material for: Safety profiling of technical lignins originating from various bioresources and conversion processes
Source: Heliyon. 2024 May 31;10(12):e32131. doi: 10.1016/j.heliyon.2024.e32131 (PMC11233868; doi:10.1016/j.heliyon.2024.e32131)
Supplement: Supplementary file 1 [file mmc1.docx]

**Safety profiling of technical lignins originating from various bioresources and conversion processes**

**T. Jayabalan, ^(1)^ P. Pandard, ^(1)^ G. Binotto, ^(1)^ J. Gomes, ^(1)^ X. Ceschini, ^(1)^ A. Aube, ^(1)^ F. Gondelle, ^(1)^  F. Pion, ^(2)^ S. Baumberger, ^(2)^ A. Jongerius, ^(3)^ R.J.A. Gosselink, ^(4)^ E. Cozzoni, ^(5)^ G. Marlair ^(1)^**

*^(1)^ Institut National de l'Environnement Industriel et des Risques (Ineris), Parc Technologique Alata, 60550 Verneuil-en-Halatte (France)*

*^(2)^ Institut Jean-Pierre Bourgin, INRAE, AgroParisTech, CNRS, Université Paris-Saclay, 78000 Versailles, France*

*^(3)^* *Avantium Chemicals B.V., Zekeringstraat 29, 1014 BV Amsterdam, The Netherlands.*

*^(4)^ Wageningen Food and Biobased Research, 6708 WG Wageningen, The Netherlands*

*^(5)^ BEES Design, Via Bargellini n. 7, 50059 Vinci, Florence, Italy*

Supplementary Information


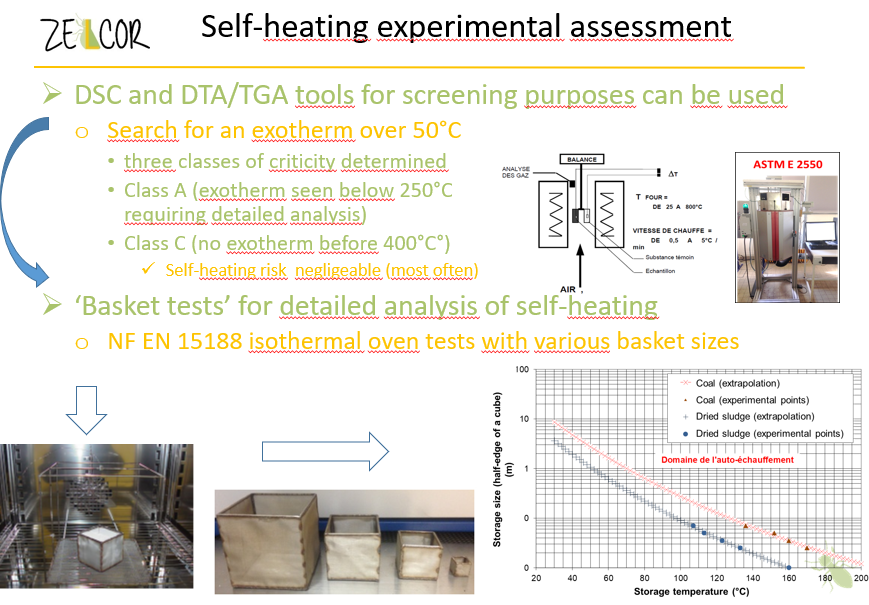


Figure S1 Guiding principles for the self-heating assessment of technical lignins
 used in the ZELCOR project (source: Zelcor webinar, July 2021)

In the screening process by use of DTA/TGA, significant exotherm leading to an increase of >50°C is taken into consideration to sort the samples according to test results into classes A (prominent risk) to C (negligible risk).

So called basket tests for full characterization of the self-heating risk were not performed in this work, although of technical interest for safety engineering purposes (towards sizing critical maximum bulk storage capacity for a given ambient temperature and maximum storage duration), since required quantities of test samples could not be supplied in the context of the ZELCOR project


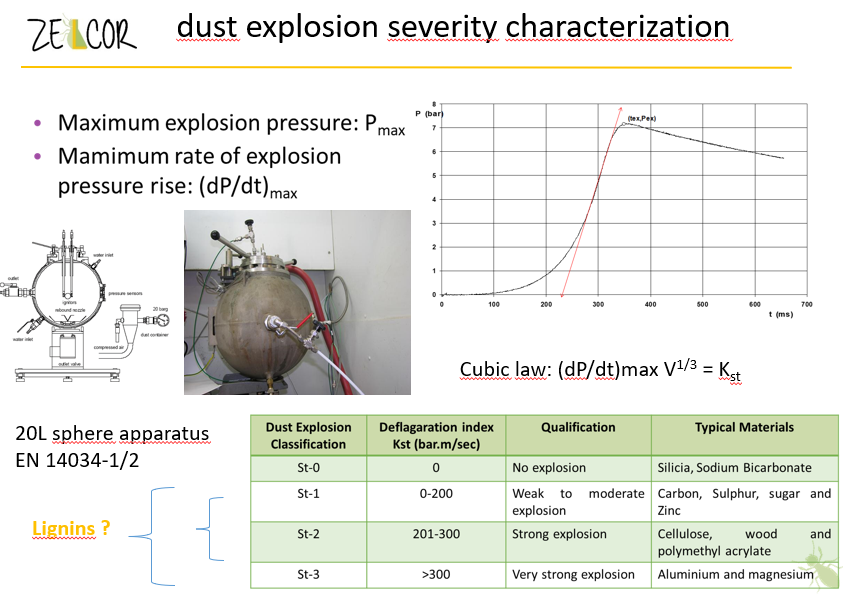


Figure S2: Schematic and photographic view of the 20l sphere used at Ineris for the assessment of dust explosion severity in the context of the ZELCOR project (source: Zelcor webinar, July 2021)


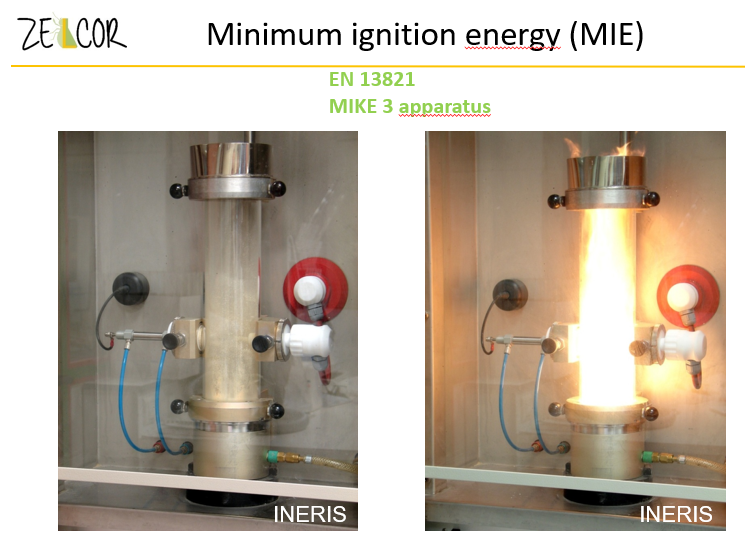


Figure S3 stand-by and in operando modes of the Mike 3 apparatus (meeting requirement of EN13821) operated by Ineris for the determination of Minimum ignition energy (MIE) of dust powder in cloud

Table ST1: Some published data outside peer review literature regarding lignin dust explosion assessment for comparison

(sources: Beck et al report: <https://www.dguv.de/ifa/publikationen/reports-download/bia-reports-1997-bis-1998/bia-report-13-97/index-2.jsp>; Hazardous Chemical Handbook (2^nd^ edition 2002): <http://ccc.chem.pitt.edu/wipf/Web/HCH.pdf>; technical lignin MSD


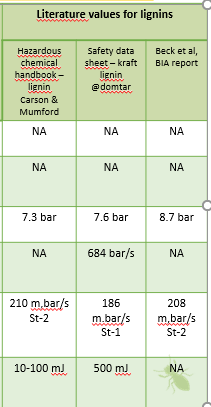

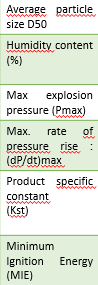


S; …)

As can be seen from table ST1, full comparison with lignin samples tested in our work is not feasible (see tables 6 to 8 on main manuscript), because of missing data on key influencing parameters in already published data. Whilst partial consistency at least is found with our results as regard order of magnitude of maximum pressure rise and to lesser extend to maximum pressure rise, more discrepancy is observed as regard minimum MIE values (50% of our samples were determined as more easily ignitable compared to displayed values in table TS1), confirming the need to relate safety profile of technical lignins to their complete physico-chemical characteristics.
